# Rehabilitation Following Multiple Limb Amputation: A Case Report

**DOI:** 10.7759/cureus.78527

**Published:** 2025-02-04

**Authors:** Sandip Dhole, Prafull K, Vandana S Daulatabad, Rohit R Gaikar, Sumedh More

**Affiliations:** 1 Physical Medicine and Rehabilitation, All India Institute of Medical Sciences, Bibinagar, Hyderabad, IND; 2 Physiology, All India Institute of Medical Sciences, Bibinagar, Hyderabad, IND; 3 Physical Medicine and Rehabilitation, All India Institute of Medical Sciences, Nagpur, Nagpur, IND; 4 Physical Medicine and Rehabilitation, All India Institute of Physical Medicine and Rehabilitation, Mumbai, IND

**Keywords:** amputation, functional progress, multidisciplinary approach, prosthesis, rehabilitation

## Abstract

Multiple limb amputations are uncommon and may result from various causes, including trauma, metabolic disorders, severe burns, purpura fulminans, and drug use. Such amputations pose substantial physical, emotional, and social challenges, necessitating a comprehensive and multidisciplinary rehabilitation approach. This report discusses the rehabilitation of a 30-year-old male patient with multiple limb amputations, including a right transradial amputation, a left transhumeral amputation, and a left transfemoral amputation, following a railway accident.

The patient was managed with a multidisciplinary strategy, incorporating stump-strengthening exercises, scar mobilization techniques, and transcutaneous electrical nerve stimulation (TENS) therapy for neuroma management, supplemented by the administration of 2% lignocaine. Prosthetic devices, including a right below-elbow cosmo-functional prosthesis, a left above-elbow cosmo-functional prosthesis with a hook as a terminal device, and a left above-knee prosthesis, were fitted. Gait training was provided to enhance mobility, independence, and reintegration into daily and occupational activities. A tailored rehabilitation program, combined with psychological counseling, addressed both physical recovery and emotional well-being. Functional progress was assessed using the Nottingham Extended Activities of Daily Living (ADL) Index, which showed a significant improvement from a score of 0 to 27 after two weeks of rehabilitation. This case underscores the importance of individualized, multidisciplinary care in achieving optimal functional outcomes and quality of life for patients with multiple limb amputations.

## Introduction

Amputation is the surgical removal of an external body part, most commonly a limb or a portion of it, performed as a form of medical treatment [1]. Multiple limb amputations are rare and may result from various causes, including metabolic disorders such as cryoglobulinemia [2], severe burns [3-5], purpura fulminans [6-8], and drug use, particularly cocaine [9]. Multiple limb amputations, particularly those involving at least one upper extremity, are extremely rare. The combination of an upper and lower limb amputation is even more uncommon, presenting unique challenges for therapists. Due to the rarity of these cases, there is limited guidance on the most effective treatment methods [10-14]. While many protocols designed for single-limb amputations can be applied in such cases, adjustments are necessary to address the distinct needs of individuals with multiple limb loss. Additionally, the loss of both an arm and a leg complicates the process of donning prostheses and makes traditional mobility aids, such as crutches and parallel bars, difficult to use effectively. These patients require tailored therapeutic approaches and innovative solutions to support functional independence and quality of life. Such amputations pose substantial physical, emotional, and social challenges, necessitating a comprehensive and multidisciplinary rehabilitation approach.

## Case presentation

**Figure 1:**
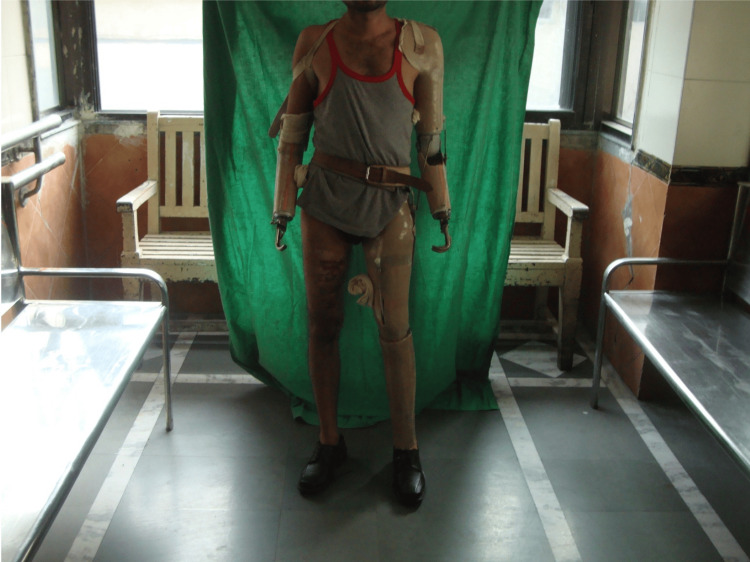
Prosthetic management

A 30-year-old male hotel supervisor presented to the All India Institute of Physical Medicine and Rehabilitation, Mumbai with multiple amputations following a railway accident 11 months prior. The patient had undergone a right transradial amputation, left transhumeral amputation, and left transfemoral amputation. Post-amputation duration was approximately 11 months. The patient was managed with a multidisciplinary strategy, incorporating stump-strengthening exercises, scar mobilization techniques, and transcutaneous electrical nerve stimulation (TENS) therapy for neuroma management, supplemented by the administration of 2% lignocaine. Prosthetic devices, including a right below-elbow prosthesis, a left above-elbow prosthesis, and a left above-knee prosthesis, were fitted as shown in Figure 1.

Gait training was provided to enhance mobility, independence, and reintegration into daily and occupational activities. A tailored rehabilitation program, combined with psychological counseling, addressed both physical recovery and emotional well-being.

**Figure 2:**
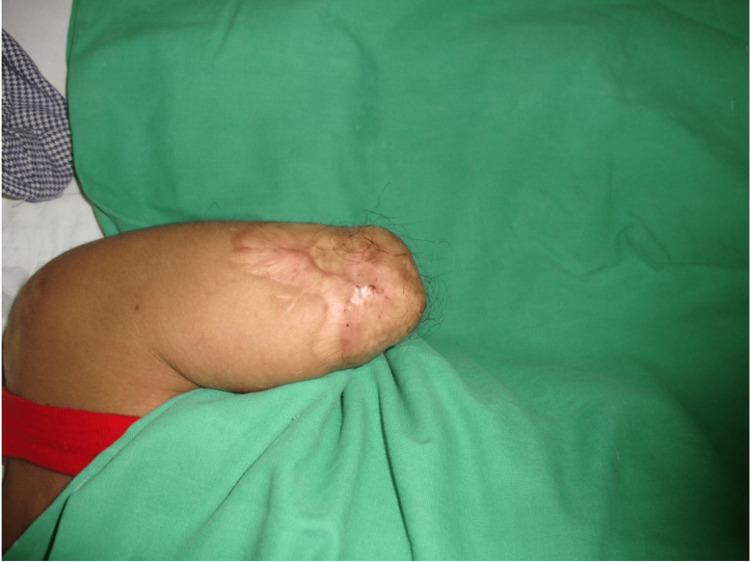
Transhumeral amputation

The patient had an above-elbow stump which was conical in shape with a bony prominence on the inferior aspect. It measured 6.5 inches in length and 8.5 inches in girth as shown below (Figure 2).

**Figure 3:**
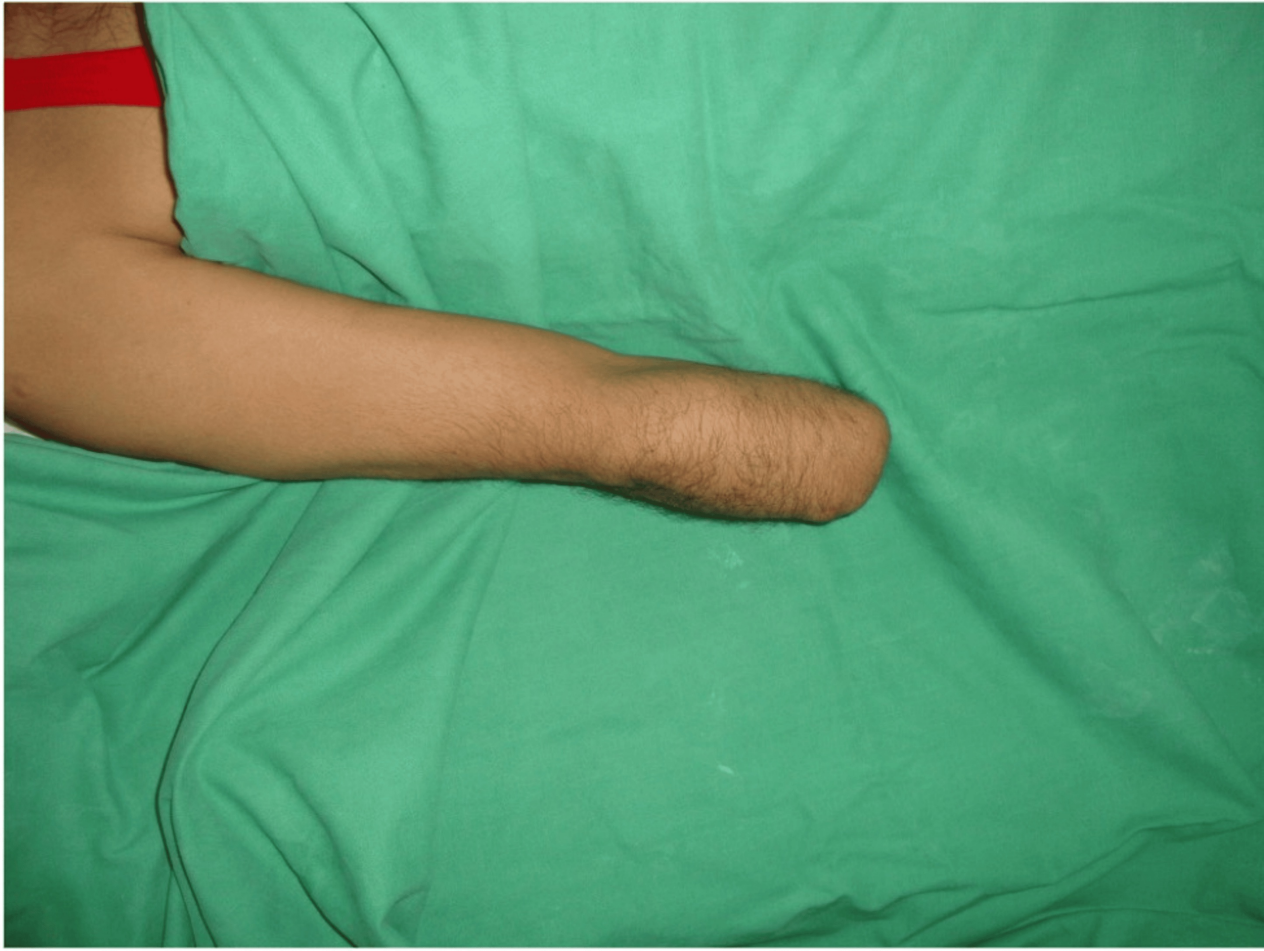
Transradial amputation

The scar was well-healed but adherent and painful. A neuroma was present, and phantom sensations were reported. Sensory function was normal, motor power was 5/5 at the shoulder joint, but abduction was restricted to 0-90 degrees. The below-elbow stump was cylindrical and bulbous. It measured 4 inches in length and 9.5 inches in girth (Figure 3).

**Figure 4:**
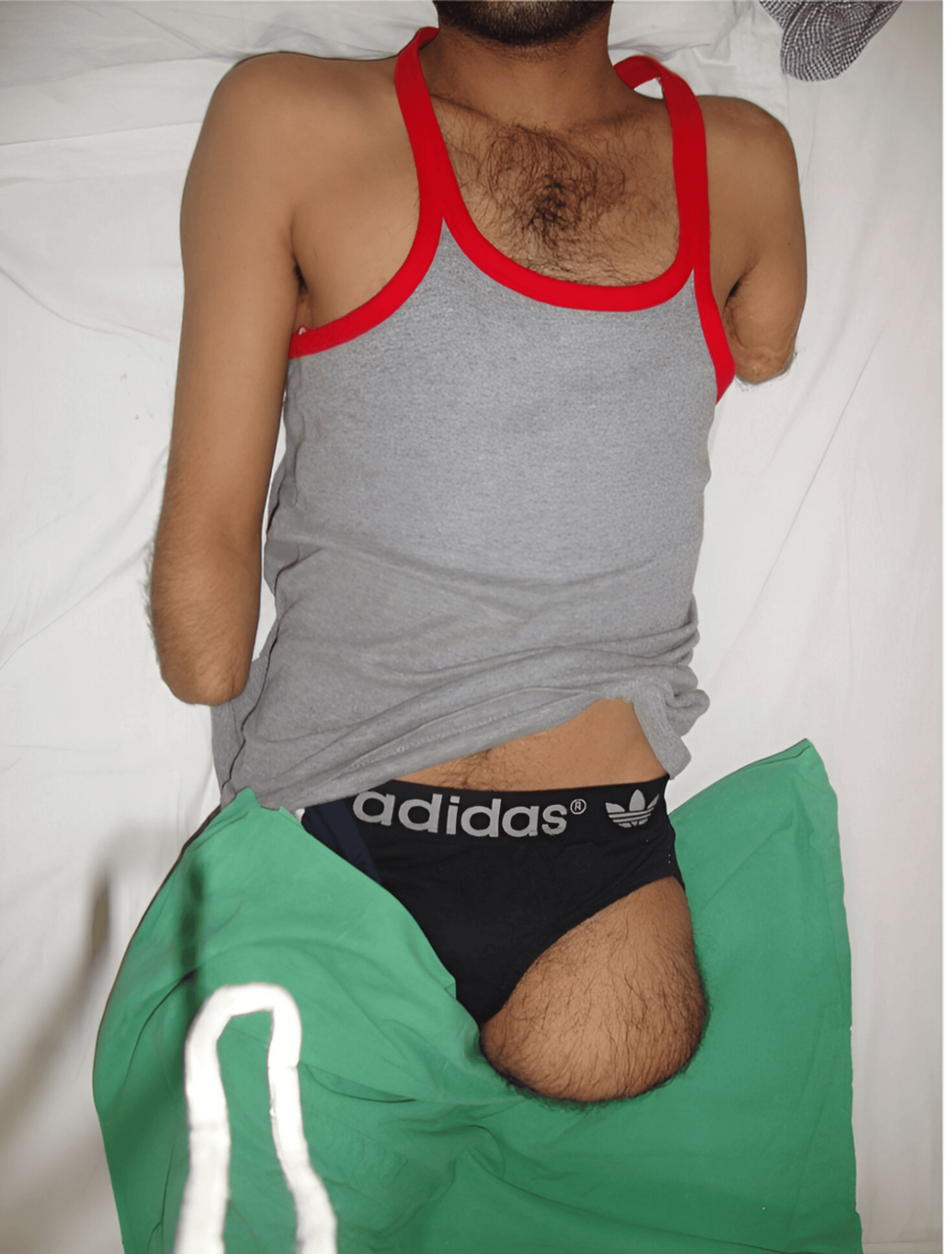
Transfemoral amputation

The scar was well-healed but centrally adherent. There was no neuroma, but phantom sensations were present. Sensory function was normal, and motor power was 5/5 at the elbow joint. The above-knee stump was cylindrical in shape, measuring 6 inches in length and 17 inches in girth as shown below (Figure 4).

The scar was well-healed and adherent. No neuroma was present, but phantom sensations were reported. Sensory function was normal, and motor power was 5/5 at the hip joint. The patient was prescribed strengthening exercises for the stumps to improve muscle strength and functionality. Trunk exercises were introduced to enhance core stability and balance. Range of motion exercises were implemented to address mobility restrictions, particularly for the above-elbow stump.

Scar mobilization techniques were employed to reduce adhesions and improve scar flexibility. For neuroma management, TENS therapy was utilized to alleviate pain and discomfort. Additionally, the patient received an injection of 2% lignocaine for local anesthetic effects and an injection of Depo-Medrol (methylprednisolone) to reduce inflammation and manage pain. For the right below-elbow stump, a functional prosthesis was given. It included a split hook for gripping, a wrist unit for adjustable positioning, and a transradial socket for a secure and comfortable fit. The prosthesis also featured a flexible elbow hinge for natural arm movement, a tricep pad for upper-arm support, and a figure-of-eight harness for suspension and control. For the left above-elbow stump, a functional prosthesis was given. It consisted of a split hook, a wrist unit for enhanced mobility, and a transhumeral socket for stability and comfort. The figure-of-eight harness provides proper suspension and control. For the left above-knee stump, a prosthesis was given. It included a pelvic belt for suspension, a hip joint without a locking mechanism for natural movement, and a quadrilateral socket for weight distribution and stump support.

The prosthesis also featured a knee joint with a manual lock for standing stability, an exo-skeletal shank for durability, and a SACH foot for reliable, shock-absorbing functionality. Functional progress was assessed using the Nottingham Extended Activities of Daily Living (ADL) Index, which showed a significant improvement from a score of 0 to 27 after two weeks of rehabilitation. The index scores revealed that with proper prosthetic and orthotic prescription and with training, rehabilitation of multi-limb amputees provides good results, drastically improving ADL activities and overall quality of life. The patient was very satisfied with the rehabilitation process he underwent. 

## Discussion

The rehabilitation of patients with multiple limb amputations poses unique challenges that require a multidisciplinary approach to address physical, functional, and emotional needs. In this case, the patient’s injuries from a railway accident had resulted in right transradial, left transhumeral, and left transfemoral amputations, which significantly impacted his mobility, independence, and psychological well-being.

Physical rehabilitation

The physical rehabilitation program emphasized stump-strengthening exercises to maintain muscle tone, improve balance, and facilitate prosthetic fitting. Stump-specific interventions, such as scar mobilization techniques, addressed adhesions and enhanced tissue flexibility. Neuroma management using TENS therapy, combined with lignocaine and methylprednisolone injections, effectively alleviated pain and discomfort, enabling smoother prosthetic adaptation.

Prosthetic rehabilitation

The prosthetic devices tailored for the patient were instrumental in restoring partial functionality. The right below-elbow prosthesis, left above-elbow prosthesis, and left above-knee prosthesis were equipped with specific features to enhance usability, stability, and comfort. Gait training further supported the patient’s reintegration into daily activities and work life, focusing on improving coordination and confidence in prosthetic use.

Psychological rehabilitation

Psychological counseling was a crucial component of the rehabilitation process. The emotional toll of multiple limb amputations can be profound, and addressing these challenges helped the patient adapt to his new reality. Counseling sessions facilitated coping strategies and promoted emotional resilience, which were critical for overall recovery.

Functional outcomes

The Nottingham Extended ADL Index demonstrated a significant improvement in the patient’s functional abilities, reflected by an increase in the score from 0 to 27 within two weeks. This rapid progress underscores the effectiveness of a well-structured and individualized rehabilitation program in optimizing outcomes for patients with complex amputations.

Clinical implications 

This report highlights the importance of a holistic, multidisciplinary approach to managing multiple limb amputations. The integration of physical therapy, prosthetic fitting, neuroma management, and psychological support plays a pivotal role in enhancing the patient’s quality of life and functional independence. Early and coordinated rehabilitation interventions can significantly reduce the long-term physical and emotional burden associated with such injuries.

## Conclusions

The rehabilitation of a patient with multiple limb amputations requires a tailored and multidisciplinary strategy to address the multifaceted challenges posed by these injuries. In this case, the combination of stump exercises, pain management techniques, prosthetic fitting, and psychological counseling resulted in significant functional and emotional recovery. The marked improvement in the Nottingham Extended ADL Index highlights the potential for individualized care to restore independence and improve the overall quality of life. This report underscores the critical role of comprehensive rehabilitation in optimizing outcomes for patients with complex amputation scenarios.
